# Gut Microbiota Mediates the Protective Effects of Dietary Capsaicin against Chronic Low-Grade Inflammation and Associated Obesity Induced by High-Fat Diet

**DOI:** 10.1128/mBio.00470-17

**Published:** 2017-05-23

**Authors:** Chao Kang, Bin Wang, Kanakaraju Kaliannan, Xiaolan Wang, Hedong Lang, Suocheng Hui, Li Huang, Yong Zhang, Ming Zhou, Mengting Chen, Mantian Mi

**Affiliations:** a Research Center for Nutrition and Food Safety, Institute of Military Preventive Medicine, Third Military Medical University, Chongqing Key Laboratory of Nutrition and Food Safety, Chongqing Medical Nutrition Research Center, Chongqing, People’s Republic of China; b Laboratory for Lipid Medicine and Technology, Department of Medicine, Massachusetts General Hospital and Harvard Medical School, Boston, Massachusetts, USA; Inter American University of Puerto Rico Metropolitan Campus; New York University School of Medicine

**Keywords:** capsaicin, chronic low-grade inflammation, gut barrier, gut microbiota, metabolic endotoxemia

## Abstract

Metabolic endotoxemia originating from dysbiotic gut microbiota has been identified as a primary mediator for triggering the chronic low-grade inflammation (CLGI) responsible for the development of obesity. Capsaicin (CAP) is the major pungent bioactivator in chili peppers and has potent anti-obesity functions, yet the mechanisms linking this effect to gut microbiota remain obscure. Here we show that mice fed a high-fat diet (HFD) supplemented with CAP exhibit lower levels of metabolic endotoxemia and CLGI associated with lower body weight gain. High-resolution responses of the microbiota were examined by 16S rRNA sequencing, short-chain fatty acid (SCFA) measurements, and phylogenetic reconstruction of unobserved states (PICRUSt) analysis. The results showed, among others, that dietary CAP induced increased levels of butyrate-producing *Ruminococcaceae* and *Lachnospiraceae*, while it caused lower levels of members of the lipopolysaccharide (LPS)-producing family S24_7. Predicted function analysis (PICRUSt) showed depletion of genes involved in bacterial LPS synthesis in response to CAP. We further identified that inhibition of cannabinoid receptor type 1 (CB_1_) by CAP also contributes to prevention of HFD-induced gut barrier dysfunction. Importantly, fecal microbiota transplantation experiments conducted in germfree mice demonstrated that dietary CAP-induced protection against HFD-induced obesity is transferrable. Moreover, microbiota depletion by a cocktail of antibiotics was sufficient to block the CAP-induced protective phenotype against obesity, further suggesting the role of microbiota in this context. Together, our findings uncover an interaction between dietary CAP and gut microbiota as a novel mechanism for the anti-obesity effect of CAP acting through prevention of microbial dysbiosis, gut barrier dysfunction, and chronic low-grade inflammation.

## IMPORTANCE

Metabolic endotoxemia due to gut microbial dysbiosis is a major contributor to the pathogenesis of chronic low-grade inflammation (CLGI), which primarily mediates the development of obesity. A dietary strategy to reduce endotoxemia appears to be an effective approach for addressing the issue of obesity. Capsaicin (CAP) is the major pungent component in red chili (genus *Capsicum*). Little is known about the role of gut microbiota in the anti-obesity effect of CAP. High-throughput 16S rRNA gene sequencing revealed that CAP significantly increased butyragenic bacteria and decreased LPS-producing bacteria (e.g., members of the S24-7 family) and LPS biosynthesis. By using antibiotics and microbiota transplantation, we prove that gut microbiota plays a causal role in dietary CAP-induced protective phenotype against high-fat-diet-induced CLGI and obesity. Moreover, CB_1_ inhibition was partially involved in the beneficial effect of CAP. Together, these data suggest that the gut microbiome is a critical factor for the anti-obesity effects of CAP.

## INTRODUCTION

Accumulating evidence suggests that chronic low-grade inflammation (CLGI) is central to the pathogenesis of obesity (1, 2), which is considered a global public health problem (3), and is linked with several complications, including insulin resistance (2). Systemic CLGI is identified by elevated circulating levels of inflammatory cytokines, such as tumor necrosis factor alpha (TNF-α), interleukin-1β (IL-1β), and IL-6, which act as molecular mediators and are responsible for the progression of the response to a systemic level encompassing multiple organs (2). It is well known that systemic CLGI damages pancreatic beta cells, disrupts insulin action, and mediates glucose intolerance in obesity (4).

Metabolic endotoxemia has been shown to be the primary contributor to the pathogenesis of CLGI, characterized by increased plasma bacterial lipopolysaccharide (LPS) levels, which are believed to originate from bacteria of the Gram-negative gut microbiome that spill into the systemic circulation as a direct result of gut barrier dysfunction (5). The binding of LPS with Toll-like receptor-4 (TLR4) leads to the increased expression of proinflammatory cytokines (6). Diet is the most important factor that determines the gut microbiota composition. The high-fat-diet (HFD)-induced obesity-promoting altered microbiome and the resultant gut barrier disintegration have been implicated as key factors facilitating metabolic endotoxemia (5, 6).

Altered gut microbiota composition (e.g., increased abundance of LPS-producing bacteria) (7) and/or function (e.g., changes in LPS biosynthesis and short-chain fatty acid [SCFA] production) (8, 9) are key factors affecting gut barrier integrity. For example, butyrate, propionate, and acetate are SCFAs derived from the bacterial degradation of complex polysaccharides in the gut (9). They have important metabolic roles, with butyrate acting as a metabolic substrate for colonic epithelial cells. The functions mediated by butyrate that are pertinent to the pathogenesis of obesity include its role in the maintenance of intestinal epithelial integrity, thereby preventing the occurrence of HFD-induced metabolic endotoxemia (10). Thus, many efforts have been made to modify metabolic endotoxemia through dietary intervention, which could be a promising way to prevent obesity and related diseases.

In addition to altered gut microbiota and function, cannabinoids modulate intestinal permeability through expression of cannabinoid receptor type 1 (CB_1_) (11). Although the expression of CB_1_ is traditionally believed to be limited to the central nervous system, recent evidence suggests that gastrointestinal expression also occurs (12). Interestingly, administration of the CB_1_ antagonist in humans is accompanied by decreased gut permeability (11) and body weight (13), and treatment with a CB_1_ agonist (HU-210) enhanced LPS-induced decreases in the expression of mRNA for gut tight junction markers such as occludin and zonula occludens-1 (14).

Chili peppers are increasingly being used in food and are very popular worldwide. Capsaicin (CAP) is the major pungent component in red chili (genus *Capsicum*) that provides flavor to food without increasing the number of calories (15). Growing evidence indicates that CAP could improve obesity and related comorbidities, suggesting that it could be a new promising therapeutic strategy (16). Previous studies have demonstrated that dietary CAP can reduce HFD-induced increase in body weight and glucose metabolism abnormalities (17–19). However, the mechanisms that underlie the anti-obesity functions remain obscure. Considering the central role of metabolic endotoxemia in the development of CLGI, we hypothesize that the anti-obesity effect of dietary CAP is due to the prevention of microbial dysbiosis-induced gut barrier dysfunction and subsequently improved metabolic endotoxemia through altering the gut microbiota and inhibiting the expression of gut CB_1_ receptor. Our findings uncover an interaction between dietary CAP and gut microbiota and provide a novel mechanism for the anti-obesity effects of CAP through prevention of metabolic endotoxemia and gut barrier dysfunction.

## RESULTS

### Dietary CAP reduces metabolic endotoxemia and systemic low-grade inflammation.

**FIG 1:**
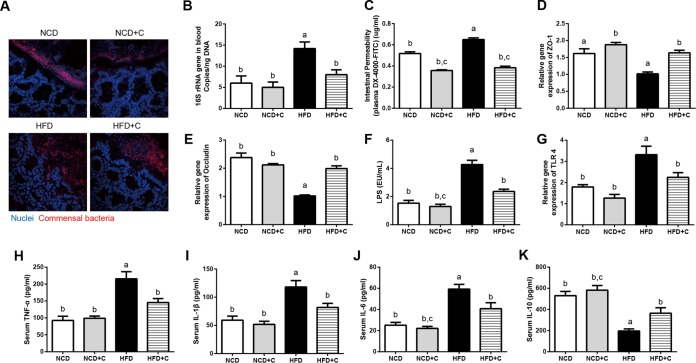
Dietary CAP reduces HFD-induced metabolic endotoxemia and systemic chronic low-grade inflammation (CLGI). Male WT mice (*n =* 6/group) were fed either NCD±C or HFD±C for 12 weeks. Blood and different tissue samples were collected and subjected to various analyses. Shown are markers of bacterial translocation and metabolic endotoxemia, such as bacterial invasion into colonic mucosa visualized by FISH (16S rRNA genes of all bacteria [red] and nuclei [blue]) (A), total bacterial DNA load (universal 16S rRNA gene copies) in whole-blood samples (B), gut permeability to FITC-dextran (C), mRNA levels of tight junction proteins ZO-1 (D) and occludin (E), plasma levels of LPS (F) and its receptor (TLR4) in epididymal white adipose tissue at the mRNA level (G), and the markers of systemic CLGI, such as plasma TNF-α (H), IL-1β (I), IL-6 (J), and IL-10 (K). Data are expressed as means ± standard errors of the means (SEM). Data with different superscript letters are significantly different (*P* < 0.05) by one-way ANOVA with a *post hoc* Bonferroni’s multiple-comparison test.

High-fat-diet (HFD)-induced metabolic endotoxemia and systemic CLGI play key roles in the pathogenesis of obesity (2). To investigate the impact of CAP on metabolic endotoxemia and systemic CLGI (plasma TNF-α, IL-1β, IL-6, and IL-10), mice were fed either a normal chow diet (NCD) or an HFD with or without CAP supplementation for 12 weeks. As shown in Fig. 1, dietary CAP reduced HFD induced elevation of the markers of bacterial translocation (bacterial invasion of epithelium and bacterial DNA in the systemic circulation) and metabolic endotoxemia (intestinal permeability to macromolecules like fluorescein isothiocyanate [FITC]-dextran, gut tight junction protein expression, and plasma LPS). In detail, bacterial invasion of epithelium was seen by fluorescence *in situ* hybridization (FISH) with a universal 16S rRNA gene probe. A dramatic increase in the number of bacteria in direct contact with the colon intestinal epithelial surface seen in the HFD group was reduced by CAP (Fig. 1A). Bacterial invasion of epithelium has been associated with bacterial translocation from the intestine and subsequently impaired metabolic homeostasis (20). Therefore, we determined the levels of bacterial DNA in blood using quantitative PCR (qPCR). In agreement with FISH results, we found that the bacterial DNA load was significantly lower in blood samples from mice fed HFD with CAP for 12 weeks (Fig. 1B). In addition, we found that the CAP-treated animals were protected from the increase in intestinal permeability and downregulation of intestinal tight junction proteins (ZO-1 and occludin) that occurs in response to an HFD (Fig. 1C to E). Excessive LPS production due to altered gut microbiota leads to metabolic endotoxemia (passage of LPS to systemic circulation) through leaky gut (5), so we next measured the plasma LPS levels by *Limulus* amebocyte lysate (LAL) chromogenic endpoint assay. Consistently, we found significantly lower plasma LPS with CAP treatment (Fig. 1F). Circulating LPS causes CLGI, and adipose tissue (AT) is the major organ that releases markers of CLGI in response to LPS (21). Lower expression of TLR4, which is a receptor for LPS, in the AT also supports the presence of lower LPS in the group treated with HFD plus CAP (HFD+C) compared to the group treated with HFD alone (Fig. 1G). Accordingly, the increased levels of markers of systemic CLGI (TNF-α, IL-1β, and IL-6) induced by the HFD were decreased by CAP (Fig. 1H to J), while levels of the anti-inflammatory cytokine IL-10 were significantly increased (Fig. 1K). Furthermore, markers of obesity (body weight and excessive visceral adiposity measured by fat pad weight) and insulin resistance (glucose intolerance measured by oral glucose tolerance tests [GTTs]) (see Fig. S1 in the supplemental material) were similarly improved by CAP. These results demonstrate that dietary CAP reduces HFD-induced metabolic endotoxemia and CLGI and associated obesity.

10.1128/mBio.00470-17.2 FIG S1  CAP reduces body weight, glucose intolerance, and systemic low-grade inflammation. (A) Time course of body weight from mice fed a normal chow diet (NCD) and a high-fat diet (HFD), both with and without CAP supplementation over 12 weeks (*n =* 6/group). The effects of dietary CAP on body weight (B), food intake (C), glucose tolerance (D), and subcutaneous (E) and epididymal (F) adipose fat tissue weight are shown. Data are expressed as means ± SEM. Data with different superscript letters are significantly different (*, *P* < 0.05) using ANOVA with a *post hoc* Bonferroni’s multiple-comparison test. Download FIG S1, PDF file, 0.3 MB. Copyright © 2017 Kang et al. 2017 Kang et al. This content is distributed under the terms of the Creative Commons Attribution 4.0 International license.

### Dietary CAP beneficially alters the gut microbiota.

**FIG 2:**
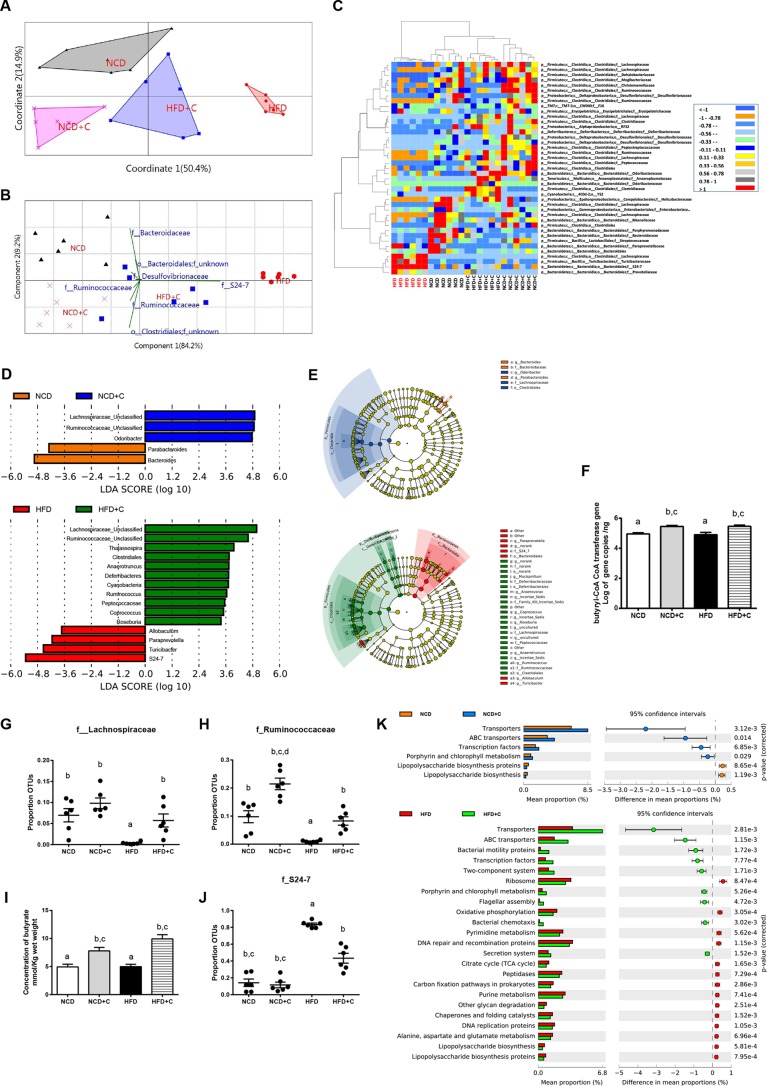
Dietary CAP beneficially alters the gut microbiota. Male WT mice (*n =* 6/group) were fed either NCD±C or HFD±C for 12 weeks. Cecal contents were subjected to 16S rRNA gene sequencing and qPCR analysis. (A) Principal-coordinate analysis based on the Bray-Curtis similarity index with the PERMANOVA significance test. (B) Similarity percentage (SIMPER) analysis, which was used to identify the specific genera with the greatest contribution to the differences observed between the groups, followed by principal-component analysis (variance-covariance type) showing the top 7 operational taxonomic unit (OTU) scores included as vectors. The magnitude and direction correspond to the weights. (C) Hierarchical clustering with a heat map shows the relative abundance of representative OTUs (those with greatest difference between the HFD and HFD+C group means from each family) selected for *P* < 0.05, obtained with differential expression analysis of the four groups. The OTUs are shown as phylum, class, order, and family. (D) Linear discriminant analysis (LDA) scores derived from LEfSe analysis, showing the biomarker taxa (LDA score of >2 and a significance of *P* < 0.05 determined by the Wilcoxon signed-rank test). (E) Cladogram generated from LEfSe analysis showing the relationship between taxon (the levels represent, from the inner to outer rings, phylum, class, order, family, and genus). (F) Abundance of butyryl-CoA transferase (BCoAT) genes. (G and H) Relative abundance (RA) of short-chain fatty acid (SCFA)-producing families *Lachnospiraceae* (G) and *Ruminococcaceae* (H). (I) Fecal concentration of butyrate SCFA. (J) RA of members of the Gram-negative LPS-producing family S24-7. Data are expressed as means ± SEM. Data with different superscript letters are significantly different (*P* < 0.05) by one-way ANOVA with a *post hoc* Bonferroni’s multiple-comparison test. PICRUSt analysis shows the relative abundance of predicted microbial genes related to metabolism for four different groups based on Welch’s *t* test (*P* < 0.05). The colored circles represent 95% confidence intervals calculated using Welch’s inverted method (K).

Excessive LPS production due to elevated levels of LPS-producing bacteria (22) and hyper-intestinal permeability due to altered gut microbiota (7) and microbiota-derived metabolites (23) have been shown as major causes for the development of metabolic endotoxemia. To assess the impact of dietary CAP on gut microbiota, we analyzed the composition, abundance, and function of gut microbiota by high-throughput sequencing of the 16S rRNA gene V3 and V4 regions of the cecal contents of these mice. To reveal the relationships of the microbial communities hosted by individuals differing by CAP treatment, we performed several analyses describing the differences in bacterial community compositions and structures between individuals. To evaluate aspects of bacterial diversity that may be influenced by CAP treatment, we first applied measures of β diversity (similarities or differences in communities between individuals). To measure community similarity, we used the Jaccard, Bray-Curtis, and UniFrac indices, which are classical ecological β diversity measures based on the ratio of shared and unique species relative to the total number of species present between two communities, respectively. This analysis revealed a significant impact of CAP treatment overall (permutational multivariate analysis of variance [PERMANOVA], Bray-Curtis, 999 mutations, *F* = 10.93, *P* = 0.001; Jaccard, 999 mutations, *F* = 3.9, *P* = 0.001; unweighted UniFrac, 999 mutations, *F* = 4.67, *P* = 0.001) and also between groups (Fig. 2A; see Fig. S2 in the supplemental material). Differences in bacterial community structures are also apparent in principal-coordinate analyses (PCoA) of these β diversity indices. PCoA revealed a distinct clustering of microbiota composition between the CAP-supplemented HFD (HFD+C), HFD, and NCD/CAP-supplemented NCD (NCD+C) groups along the primary ordination axis (axis 1), which explains 50.4% of the variation in the data (Fig. 2A). On one end of the axis, the HFD group stand alone, and HFD+C is in the middle, while the NCD/NCD+C cluster is on the opposite end. Next, taxa that are primarily responsible for an observed difference between groups were analyzed using the similarity percentage (SIMPER) test, which reveals 7 taxa. Their contributions to groups are analyzed using principal component (variance-covariance type) analysis (PCA) (Fig. 2B). Accordingly, Gram-negative LPS-producing members of the S24_7 and SCFA-producing *Ruminococcacea* families are primarily responsible for HFD- and CAP-treated groups, respectively. Furthermore, the relative abundance of taxa, which showed *P* value of <0.05 by differential expression analysis (nonparametric ANOVA with false discovery rate [FDR] correction), was expressed as a heat map (Fig. 2C) including hierarchical clustering (HCN). HCN is a clustering technique for graphically summarizing the intersample relationships in the form of a dendrogram. HCN also clearly separated the HFD samples as a single cluster from the other 3 groups, which form three clusters within a clade. In addition, biomarker analysis using linear discriminant analysis (LDA) effect size (LEfSe) (Fig. 2D) and a cladogram (Fig. 2E) generated from LEfSe analysis indicated that the members of the LPS-producing family S24_7 were increased in the HFD group, while SCFA (e.g., butyrate)-producing *Ruminococcaceae* and *Lachnospiraceae* were increased in the CAP-treated group (Fig. 2D and E). Because various high-throughput microbiome analyses emphasize the main effects of CAP on SCFA-producing bacteria, we quantified butyryl coenzyme A (CoA) transferase (BCoAT) genes using qPCR. Interestingly, we found that the gene copies were higher in the CAP-treated HFD group (Fig. 2F). Consistently, the relative abundances (RAs) of SCFA-producing bacteria measured by both sequencing and qPCR were significantly higher in CAP-treated groups (Fig. 2G and H; see Fig. S3A and B in the supplemental material). Moreover, the fecal SCFA analysis revealed a dramatic increase in butyrate in CAP-treated groups (Fig. 2I; Fig. S3C and D). Butyrate, which is a major SCFA produced by *Ruminococcacaea* (24), prevents metabolic endotoxemia (23) by strengthening the gut barrier. Likewise, RA of the LPS-producing S24_7 family was significantly higher in HFD and vice versa in CAP-treated groups (Fig. 2J). Next, to study the potential function of gut microbiota in different groups, LEfSe was applied to the relative abundance of KEGG pathways predicted by phylogenetic reconstruction of unobserved states (PICRUSt) (25). Notably, the biomarkers with significant discriminative power were the “lipopolysaccharide biosynthesis proteins” and “lipopolysaccharide biosynthesis pathways,” which were significantly lower in CAP-treated groups than in those fed on HFD alone (Fig. 2K).

10.1128/mBio.00470-17.3 FIG S2  CAP beneficially alters gut microbiota. (A) PCoA analysis using the Jaccard index. (B) PCoA analysis using the unweighted UniFrac distance. PC1, principal coordinate 1. Download FIG S2, PDF file, 0.6 MB. Copyright © 2017 Kang et al. 2017 Kang et al. This content is distributed under the terms of the Creative Commons Attribution 4.0 International license.

10.1128/mBio.00470-17.4 FIG S3  CAP alters the abundance of cecal butyrate-producing bacteria and fecal SCFA concentrations. (A and B) qPCR analysis showing the abundance (gene copies) of *Clostridium* cluster IV (A) and *Clostridium* cluster XIVa (B). (C and D) Fecal concentrations of acetate (C) and propionate (D). Data are expressed as means ± SEM. Data with different superscript letters are significantly different (*P* < 0.05) using one-way ANOVA with a *post hoc* Bonferroni’s multiple-comparison test. Download FIG S3, PDF file, 0.2 MB. Copyright © 2017 Kang et al. 2017 Kang et al. This content is distributed under the terms of the Creative Commons Attribution 4.0 International license.

Although β diversity measures describe aspects of community structure between groups, they do not reveal the similarities or differences in communities within a group (i.e., α diversity). We next applied measures of α diversity, which describe species composition in one specific habitat of interest and can be informative of community functioning (26). Because microbial communities are highly diverse and are often poorly amenable to the diversity measures commonly used in community ecology (27), we used several different measures focusing on different aspects of community assembly, including species richness and abundance. Chao1 (species richness), the abundance-based coverage estimator (ACE), and the Shannon index (species richness) showed a trend of increase when diet was supplemented with CAP (see Fig. S4 in the supplemental material). Together, these data indicate that CAP beneficially alters the gut microbiota and mainly prevents HFD-induced reduction of butyrate-producing bacteria and elevation of Gram-negative LPS-producing bacteria and associated LPS biosynthesis.

10.1128/mBio.00470-17.5 FIG S4  CAP beneficially alters gut microbiota. Shown are rarefaction curves of sequencing samples (A) and α-diversity (B) of gut microbial communities assessed by Chao1, ACE, and the Shannon α diversity index, respectively. Data are presented as median with interquartile range. Download FIG S4, PDF file, 0.4 MB. Copyright © 2017 Kang et al. 2017 Kang et al. This content is distributed under the terms of the Creative Commons Attribution 4.0 International license.

### Gut microbiota mediates the preventive effects of CAP on metabolic endotoxemia.

**FIG 3:**
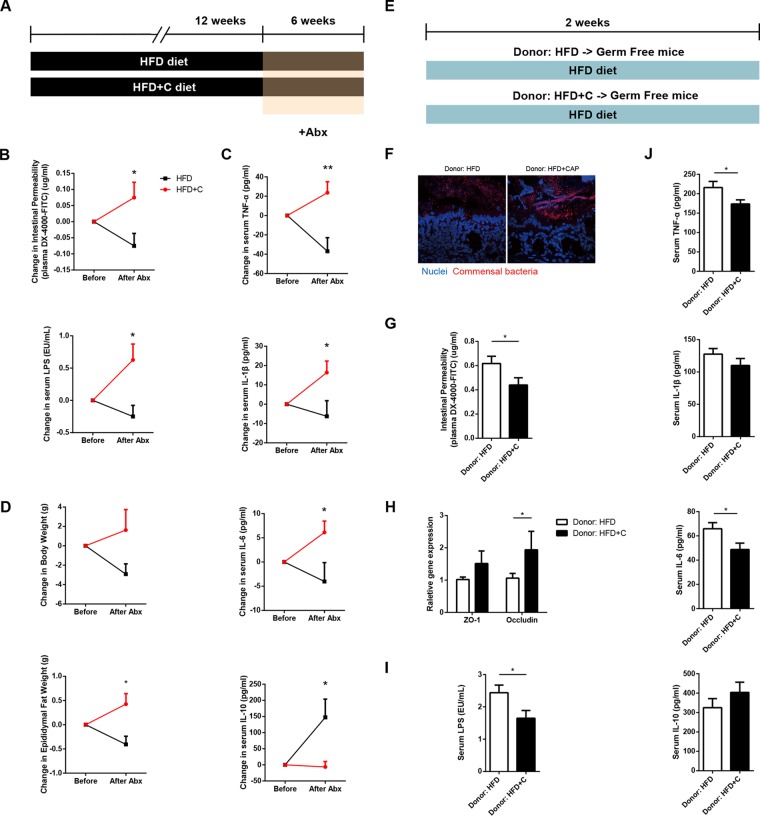
Gut microbiota mediates the preventive effects of CAP on metabolic endotoxemia. (A) Schema showing the animal groups and treatments. Male WT mice (*n =* 6/group) received either HFD±C (A) or NCD±C (Fig. S5) for 12 weeks and then were treated with a broad-spectrum antibiotic cocktail (Abx) consisting of ampicillin, vancomycin, neomycin sulfate, and metronidazole for 6 weeks to introduce microbiota depletion (A). Blood and tissue samples were subjected to various analyses, and data were analyzed before and after Abx supplementation for markers of metabolic endotoxemia (B), systemic chronic low-grade inflammation (CLGI) (TNF-α, IL-1β, IL-6 and IL-10) (C), and obesity (D). (E) Schema showing the germfree mice that received microbiota transplanted from mice treated with HFD±C for 12 weeks. Two weeks after transplantation, blood and tissue samples were subjected to various analyses, such as FISH for markers of bacterial translocation, metabolic endotoxemia, and bacterial invasion of epithelium (F), gut permeability (G), intestinal tight junction proteins (H), plasma levels of LPS (I), and plasma levels of markers of systemic CLGI (J). Data are expressed as means ± SEM, and statistical analysis was performed using ANOVA with a *post hoc* Bonferroni’s multiple-comparison test or unpaired two-tailed Student’s *t* test.

Metabolic endotoxemia is commonly derived from dysbiotic gut microbiota, so we next determined whether gut microbiota is necessary for the beneficial effects of CAP on metabolic endotoxemia using antibiotic treatment and fecal microbiota transplantation (FMT) experiments. We used a cocktail of broad-spectrum antibiotics (Abx [ampicillin, metronidazole, neomycin, and vancomycin]) to create groups of macroscopically germfree (GF) mice (7), which were fed NCD and HFD in both the absence and presence of CAP supplementation (Fig. 3A). Interestingly, when comparing the data gathered before and after treatment with antibiotics, the differences observed between HFD and HFD+C groups in markers of metabolic endotoxemia (Fig. 3B), inflammation (Fig. 3C), and obesity (Fig. 3D; see Fig. S5 in the supplemental material) were eliminated. These findings suggest that the gut microbiota largely mediates the preventive effects of CAP on metabolic endotoxemia. More specifically, the HFD+C group may carry a lower abundance of LPS-producing bacteria.

10.1128/mBio.00470-17.6 FIG S5  Gut microbiota mediates the beneficial effects of CAP on chronic inflammation and obesity. WT mice (*n =* 6/group) received HFD±C and NCD±C for 12 weeks and then were treated with antibiotic (Abx) cocktail for 6 weeks. Data were analyzed before and after Abx supplementation. (A and B) HFD versus HFD+C. Shown are markers of obesity and insulin resistance. (C to H) NCD versus HFD. Shown are metabolic endotoxemia (ME) (C and D), chronic low-grade inflammation (CLGI) (E), and obesity/insulin resistance (F and H). (I to N) NCD versus NCD+C. Shown are ME (I and J), CLGI (K), and obesity/insulin resistance (L to N). Data are expressed as means ± standard errors (SE). *, *P* < 0.05, **, *P* < 0.01, and ***, *P* < 0.001, by unpaired two-tailed Student’s *t* test. Download FIG S5, PDF file, 0.6 MB. Copyright © 2017 Kang et al. 2017 Kang et al. This content is distributed under the terms of the Creative Commons Attribution 4.0 International license.

To further clarify whether the gut microbiota plays a causal role, we transplanted the fecal microbiota of mice fed HFD or HFD+C for 12 weeks into recipient GF mice and then fed both groups of recipient mice a high-fat diet for 2 weeks (Fig. 3E). Once colonized, the GF mice are maintained as gnotobiotic by housing them in sterile isolators to avoid cross contamination with other bacteria or fungi (see Fig. S6A in the supplemental material). Strikingly, markers of bacterial translocation (Fig. 3F; Fig. S6B), metabolic endotoxemia (Fig. 3G to I), and systemic CLGI (Fig. 3J) were significantly lower in GF mice that received microbiota from the CAP-treated HFD group than in GF mice that received HFD donor microbiota. As expected, lower TLR4 expression in the AT (Fig. S6C) and higher fecal SCFA levels, especially butyrate and acetate, were found in GF mice treated with HFD+C without microbiota transplant (Fig. S6D). Although there were no differences in body weights and food intakes between these two groups (Fig. S6E and F), the recipients of microbiota from the mice consuming an HFD with CAP supplementation exhibited an improvement in glucose tolerance and fat accumulation (Fig. S6G and H). Combined, these findings suggest that the beneficial effects of CAP on metabolic endotoxemia and obesity were largely mediated by modulation of the gut microbiota.

10.1128/mBio.00470-17.7 FIG S6  Anti-obesity effects of CAP microbiota are transferable to germfree mice. (A) Gnotobiotic isolator. (B to H) Bacterial DNA load in blood (B), adipose tissue TLR4 mRNA levels (C), fecal SCFA concentrations (D), body weight (E), food intake (F), glucose tolerance (G), and fat accumulation (H) of germfree mice 14 days following transplantation of microbiota from HFD-fed mice with or without CAP supplementation. Data are expressed as means ± SEM. *, *P* < 0.05, and **, *P* < 0.01, by unpaired two-tailed Student’s *t* test. Download FIG S6, PDF file, 0.2 MB. Copyright © 2017 Kang et al. 2017 Kang et al. This content is distributed under the terms of the Creative Commons Attribution 4.0 International license.

### CB_1_ receptor inhibition is involved in the beneficial effects of CAP.

**FIG 4:**
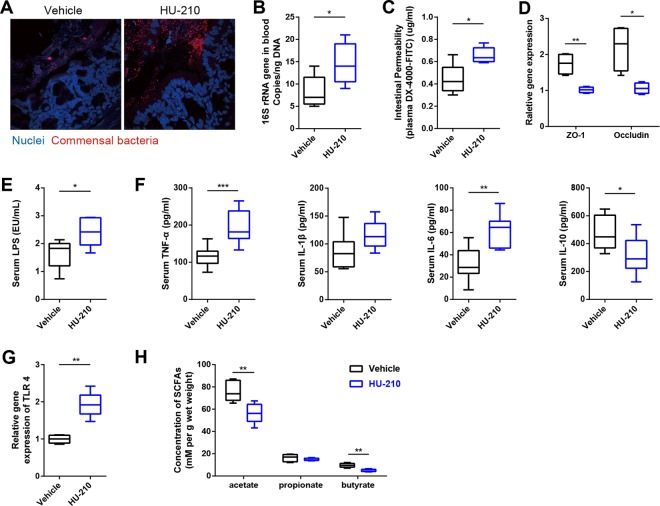
CB_1_ receptor inhibition is involved in the beneficial effects of CAP. WT mice were fed HFD+C for 12 weeks, and then HFD+C mice were divided into two treatment groups (vehicle versus HU-210). A CB_1_-specific agonist (HU-210) or vehicle was orally administered for 4 weeks (*n =* 6/group). Activation of CB_1_ eliminated the beneficial effects of CAP treatment on markers of bacterial translocation and metabolic endotoxemia, such as those shown by FISH analysis (A), whole-blood total bacterial DNA load (B), gut permeability (C), intestinal tight junction proteins (D), and plasma levels of LPS (E), in addition to chronic inflammation markers, such as plasma levels of inflammatory cytokines (TNF-α, IL-1β, IL-6, and IL-10) (F), adipose tissue TLR4 mRNA levels (G), and fecal SCFA concentrations (H). Data are expressed as means ± SEM. *, *P* < 0.05, **, *P* < 0.01, and ***, *P* < 0.001, by unpaired two-tailed Student’s *t* test.

The endocannabinoid system, particularly the cannabinoid (CB) receptor in the intestine, links the gut microbiota with metabolic endotoxemia and CLGI (28). Next, we examined the CB_1_ and CB_2_ receptors at the mRNA level in the colon. CB_1_ receptor expression was significantly lower in the HFD+C mice, whereas there were no differences in the expression of CB_2_ between these groups (see Fig. S7A in the supplemental material), so we hypothesized that the inhibition of CB_1_ also involved in the beneficial effects of dietary CAP through microbiota. To test this hypothesis, mice were fed HFD+C for 4 weeks in the presence or absence of a CB_1_ agonist (HU-210). As we expected, the beneficial effects of CAP against HFD-induced increase of markers of bacterial translocation (Fig. 4A and B), metabolic endotoxemia (Fig. 4C to E), and CLGI (Fig. 4F) were significantly prevented in the HFD+C+HU-210 group compared to the HFD+C+vehicle group. Similarly, the TLR4 expression (Fig. 4G) in the AT was upregulated, and fecal SCFA (mainly butyrate and acetate) (Fig. 4H) production was reduced in the HFD+C+HU-210 group. Consistent with other changes, the presence of the CB_1_ receptor agonist increased the markers of obesity (weight gain and fat pad weight) and insulin resistance (oral GTT) in the HFD+C group (Fig. S7B to E). Taken together, these findings indicate CAP prevents HFD-induced upregulation of CB_1_ receptor. These inhibitory effects of CAP on CB_1_ receptor could partially explain the beneficial effects of CAP on metabolic endotoxemia and obesity.

10.1128/mBio.00470-17.8 FIG S7  CAP downregulation of the CB_1_ receptor is involved in the beneficial effects of CAP on obesity and mediates the benefits of CAP treatment. (A) CB_1_ expression among mice fed with or without CAP supplementation (A). A CB_1_-specific agonist (HU-210) was administered by orally gavage to mice fed HFD+C for 4 weeks. Activation of CB_1_ eliminated the beneficial effects of CAP treatment on body weight (B), food intake (C), oral GTT (D), and fat accumulation (E). Data are expressed as means ± SEM. *, *P* < 0.05, and **, *P* < 0.01, using the unpaired two-tailed Student’s *t* test. Data with different superscript letters are significantly different (*P* < 0.05) by ANOVA with a *post hoc* Bonferroni’s multiple-comparison test. Download FIG S7, PDF file, 0.3 MB. Copyright © 2017 Kang et al. 2017 Kang et al. This content is distributed under the terms of the Creative Commons Attribution 4.0 International license.

## DISCUSSION

**FIG 5:**
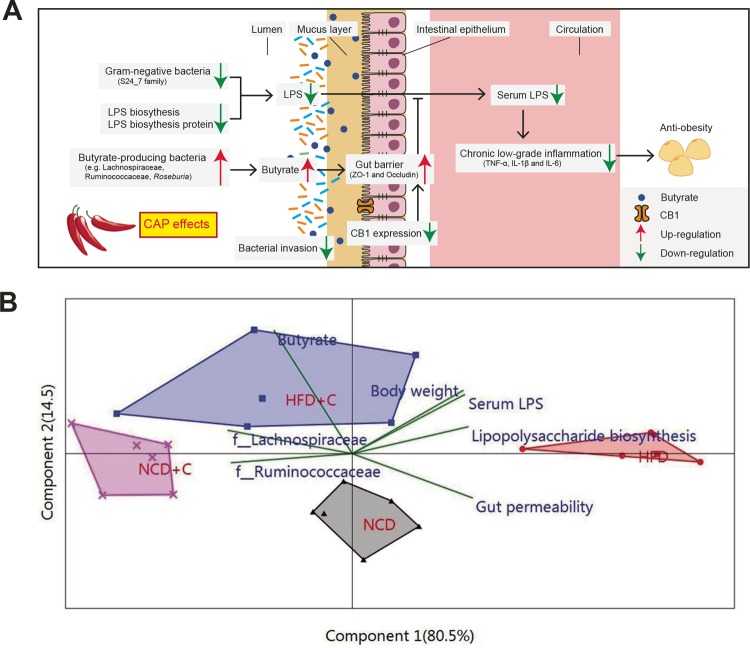
Diagram illustrating a proposed mechanism by which the interactions between dietary CAP and gut microbiome lead to a decrease in Gram-negative LPS-producing bacteria (e.g., S24-7 family) and LPS biosynthesis and an increase in butyrate-producing bacteria (e.g., *Lachnospiraceae* and *Ruminococcaceae*) and butyrate levels. These changes lower LPS production and gut permeability and downregulate CB_1_ expression, resulting in reduced markers of metabolic endotoxemia. The subsequent reduction of inflammatory cytokines leads to the suppression of chronic low-grade inflammation and obesity (A). (B) Principal-component analysis (biplot) showing the correlation between important parameters of the proposed mechanism presented above. Labels indicate the four groups. The names of the parameters are plotted as vectors according to their correlation to the first two components.

Metabolic endotoxemia originating from dysbiotic gut microbiota and impaired gut barrier integrity play a central role in the pathogenesis of chronic low-grade inflammation, an underlying factor of obesity and associated health complications (5, 6). Discovering a safe and novel means of limiting its development is urgently required for the prevention and treatment of these diseases. The present study demonstrates for the first time that anti-obesity effects of dietary CAP could be due to preventing the occurrence of HFD-induced metabolic endotoxemia and systemic CLGI (Fig. 5A). Consequently, we are able to propose a pathway-based mechanism by which dietary CAP increased the abundance of butyrate-producing bacteria, prevented HFD-induced upregulation CB_1_ expression, and reduced the expression of genes involved in LPS biosynthesis. These changes improve the gut barrier abnormalities and lower gut permeability, resulting in reduced metabolic endotoxemia (Fig. 5A). The subsequent reduction of proinflammatory cytokines leads to the prevention of CLGI and obesity (Fig. 5A). Multivariate principal component correlation analysis, including the above-mentioned findings, strongly supports the proposed mechanism (Fig. 5B). Moreover, results from antibiotic-induced depletion of gut microbiota and fecal microbiota transplantation experiments in germfree mice strongly suggest that the gut microbiota largely mediates CAP’s protection against HFD-induced obesity.

Our results demonstrate that CAP supplementation enhanced the abundance of SCFAs producing *Clostridium* clusters IV (*Ruminococcaceae*) and XIVa (*Lachnospiraceae*, including *Roseburia* spp.) in HFD-fed obese mice (Fig. 2D and E and G and H). Interestingly, our previous study also found that the levels of butyrogenic bacteria are elevated in humans after 2 weeks of CAP intervention (16). Bacterial butyrate production is a key function attributed to a “healthy” gut microbiota because of the preferred use of this SCFA as an energy source for intestinal epithelial cells (29). Previous studies have highlighted the importance of SCFAs such as acetate, propionate, and butyrate in the amelioration of chronic inflammatory diseases and the promotion of colonocyte health (29–31). SCFAs are produced from the fermentation of polysaccharides by *Lachnospiraceae*, including *Roseburia* spp. (32). In our study, fecal butyrate concentrations were positively correlated with abundance of butyrogenic *Lachnospiraceae* (Fig. 5B) in CAP-treated groups, suggesting the key role of this CAP-enhanced bacterial group in the elevated levels of butyrate with CAP intervention.

It is well-known that colonic butyrate improves gut barrier function by increasing HIF-1 through the stimulation of epithelial metabolism and enhancing the expression of proteins involved in tight junctions, thereby preventing the translocation of endotoxins produced by intestinal Gram-negative bacteria across the gut barrier (10, 23, 33–35). Besides, butyrate suppresses the production of proinflammatory cytokines, enhances anti-inflammatory IL-10 expression, and activates regulatory T cells (Treg cells), leading to the amelioration of colitis (34). The data from our antibiotics, microbiota transfer, and CB_1_ inhibition experiments also support the notion that butyrate contributes to the anti-inflammatory effect of CAP as well as its beneficial effects on the gut barrier. Combined, these results indicate that the beneficial effects of CAP against metabolic endotoxemia and associated obesity may be largely due to increases in the populations of these butyrogenic bacterial species and gut barrier-enhancing effects of butyrate.

Variations in the intestinal endocannabinoid system in response to modulation of the gut microbiota have been observed in germfree mice and in mouse models of bacterial-host interactions in colonic tissue (14). The intestinal endocannabinoid system is thought to control gut barrier function, gut permeability, and metabolic endotoxemia under obese and diabetic conditions through a CB_1_-dependent mechanism, as antagonists of CB_1_ decrease gut permeability and act as “gatekeepers” (36). Conversely, treatment with the CB_1_ receptor agonist HU-210 in mice significantly increased plasma levels of LPS and augmented the LPS-induced decreases in the expression of gut tight junction proteins at the mRNA level (14). In our study, we discovered that CAP prevented the HFD-induced upregulation of CB_1_ receptor expression because HU-210 treatment of the HFD+C group partially abolished the beneficial effects of dietary CAP on gut barrier, metabolic endotoxemia, and CLGI, indicating that CB_1_ inhibition also partially contributes to the anti-obesity effects of CAP.

In addition to the enrichment of butyrogenic bacteria and CB_1_ inhibition, we found that CAP intervention reduced the enrichment of genes involved in LPS biosynthesis and related proteins based on the predicted function by 16S rRNA sequencing and phylogenetic reconstruction of unobserved states (PICRUSt) analysis (25). Interestingly, our recent study has shown similar results with CAP intervention in human subjects (16). This would indicate the possibility that lower abundance of Gram-negative microbiota must be responsible for the low abundance of KEGG orthology (KO) belonging to the LPS biosynthesis pathway in the HFD+C group. This could be mainly due to prevention of enrichment of members of the Gram-negative S24_7 family with CAP intervention because it was the key bacterial family that largely contributes to the HFD group (Fig. 2D and J). These lipopolysaccharides are bacterium-associated molecular patterns, which act via TLR4 by promoting the inflammatory response (6).

Our study design is unique in its use of fecal microbiota transplantation in the germfree (GF) mouse model. GF mice that lack any exposure to living pathogenic or nonpathogenic microorganisms provide an attractive model to investigate the role of the composition and function of intestinal microbiota on the development of obesity (6). Given the fact that transferring the microbiota of HFD-consuming mice to GF mice caused glucose intolerance in 6 days (37) and significant changes in the luminal butyrate concentrations of recipient GF mice at day 7 post-FMT (38), it is interesting to note that the acquired changes in microbiota in response to CAP protect against HFD-induced obesity because germfree mice that received microbiota from the HFD+C group exhibited lower levels of gut permeability, plasma LPS, and markers of CLGI in 2 weeks (Fig. 3).

In conclusion, these results demonstrate dietary CAP-microbiome interactions as a novel mechanism underlying the anti-obesity effects of dietary CAP. Our study demonstrated that dietary CAP prevents HFD-induced metabolic endotoxemia and systemic CLGI by elevating cecal butyrogenic bacterium and butyrate levels, inhibiting colonic CB_1_ receptor, and reducing LPS biosynthesis (Fig. 5A). Finally, given the fact that gut dysbiosis and metabolic endotoxemia are often linked to chronic inflammatory diseases, the ability of dietary CAP to prevent these conditions confirms the potential of CAP supplementation as a therapeutic means of treating obesity.

## MATERIALS AND METHODS

### Animals.

Male wild-type (WT) mice of the C57BL/6J genetic lineage were bred in the specific-pathogen-free animal (SPF) facility of the Third Military Medical University, Chongqing, People’s Republic of China, and maintained in a temperature-controlled room (22 to 24°C) with a strictly followed 12-h light/12-h dark diurnal cycle with food and water *ad libitum*. Germfree (GF) mice were created using caesarean rederivation from existing SPF mouse lines. A total of 10 1-day-old GF mice of the C57BL/6J background were bred in the Department of Laboratory Animal Science of the Third Military Medical University and were used as recipients for the fecal microbiota transplantation. Sterile plastic film isolators, which help to avoid cross contamination with other bacteria or fungi, were used to house the mice in a completely germfree environment; this provides an environment that allowed us to conduct experiments without competing background levels of microbiota. They were given *ad libitum* access to sterilized water during the whole course of the experiment. Food and water and other sterile supplies were imported into the isolators by docking autoclaved supply cylinders to a double-door port built into the isolator wall (Fig. S6A). GF foster mice were used to breastfeed 1-day-old GF mice until weaning (which occurred at 3 weeks of age); they were then fed *ad libitum* with a sterilized normal chow diet for 5 weeks postweaning. Culture and PCR analysis of feces amplifying the 16S rRNA gene were used to routinely test the sterility of GF isolators. The animal experiments were approved by the Animal Care and Use Committee of the Third Military Medical University (Chongqing, China).

### Animal diets.

Normal chow diet (NCD [D12450B]) had 70% of kilocalories from carbohydrate, 20% of kilocalories from protein, and 10% of kilocalories from fat, for a total energy content of 3.85 kcal/g, and the high-fat diet (HFD [D12451]) had 35% of kilocalories from carbohydrate, 20% of kilocalories from protein, and 45% of kilocalories from fat, for a total energy content of 4.73 kcal/g. These diets, with or without capsaicin (Sigma, St. Louis, MO, USA) at 0.01 g CAP/100 g diet, were ordered from Research Diets, Inc. (New Brunswick, NJ, USA).

### Animal experiments.

Mice were housed in a biosafety level 2 (BSL2) room in hard-top cages with two or three mice per cage. Body weight and food intake were measured weekly. Mice were fasted for 4 h before being sacrificed, and blood was collected via cardiac puncture in all experiments unless otherwise specified.

### (i) Determination of the CAP effects on metabolic endotoxemia, chronic low-grade inflammation, and gut microbiota.

Eight-week old mice were randomly distributed into four groups (*n =* 6/group): (i) normal chow diet (NCD), (ii) NCD with 0.01% CAP (NCD+C), (iii) high-fat diet (HFD), and (iv) HFD with 0.01% CAP (HFD+C). After 12 weeks, GTT and gut permeability assays were done. Then mice were sacrificed, blood samples were aliquoted into EDTA-coated blood collection tubes, cecal contents were flash-frozen, and body fat pad weights were taken. Blood samples were snap-frozen in liquid nitrogen and stored at −80°C or centrifuged (2,500 × *g* for 15 min at 4°C) to collect plasma. Cecal contents and feces were subjected to microbiome and SCFA analysis, respectively. Analyses of markers of bacterial translocation (bacterial invasion into intestinal epithelium and bacterial DNA in the systemic circulation), metabolic endotoxemia (hyper-intestinal permeability to macromolecules like FITC-dextran, gut tight junction protein expression, and plasma LPS levels), and systemic CLGI (plasma TNF-α, IL-1β, IL-6, and IL-10) were also conducted.

### (ii) Determination of causal role of microbiota for the anti-obesity effects of CAP.

*(a) Antibiotic-induced gut microbiota depletion.* A subset of 8-week-old mice were randomly distributed into four groups (*n =* 6/group) and were fed either NCD±C or HFD±C for 12 weeks. Markers of metabolic endotoxemia, CLGI, and obesity were recorded before (at the end of the 12th week) and after treatment of these mice with a broad-spectrum antibiotic (Abx) cocktail (Sigma, USA) containing ampicillin (1 mg/ml), metronidazole (1 mg/ml), neomycin (1 mg/ml), and vancomycin (0.5 mg/ml) in their drinking water for 6 weeks. Successful depletion of gut microbiota after the antibiotic treatment was confirmed using qPCR analysis of total bacterial 16S rRNA genes (7, 39).

*(b) Fecal microbiota transplantation in germfree mice.* To colonize the guts of GF mice, fecal samples were collected from randomly chosen mice that had received HFD or HFD+C for 12 weeks since the age of 8 weeks. Fecal samples were stored at −80°C until the time of processing. The procedures for preparing the fecal samples for microbiota transplantation were performed as described in a previous study (40). In detail, fecal samples were homogenized with a mortar and pestle while submerged in liquid nitrogen. A 100-mg aliquot of the pulverized frozen material was then diluted in 1.5 ml of reduced phosphate-buffered saline (PBS) (supplemented with 0.1% resazurin [wt/vol] and 0.05% l-cysteine-HCl) in an anaerobic Coy chamber (atmosphere, 75% N_2_, 20% CO_2_, 5% H_2_) and then vortexed at room temperature for 5 min. The suspension was allowed to settle by gravity for 5 min, after which time the clarified supernatant was transferred to an anaerobic crimped tube that was then transported to the gnotobiotic mouse facility. The outer surface of the tube was sterilized by exposure for 20 min to chlorine dioxide in the transfer sleeve attached to the gnotobiotic isolator and then transferred into the isolator. A 1-ml syringe was used to recover a 200-μl aliquot of the suspension; the suspension was subsequently introduced by gavage with a flexible plastic tube into the stomach of each adult 8-week-old GF recipient. The samples were obtained shortly before colonization, immediately (within 5 min) diluted, and introduced into the GF mice within 2 h after dilution. The recipient mice were separately bred in different gnotobiotic isolators to prevent normalization of the gut microbiota. Also, the recipient mice were maintained in separate cages (five mice per cage; *n =* 5/group) within an isolator dedicated to mice colonized with the same donor microbiota. As mentioned in the previous section, mouse samples were subjected to analysis for markers of bacterial translocation, metabolic endotoxemia, and systemic CLGI and analysis of fecal SCFA levels after 2 weeks.

### (iii) Role of CB_1_ receptor for the CAP effects on metabolic endotoxemia and obesity.

To study the effects of CAP on HFD-induced upregulation of CB1, HU-210 (CB1 receptor agonist) was dissolved in a mixture of dimethyl sulfoxide (DMSO), Cremophor, and saline solution (41), and then either HU-210 or vehicle was intraperitoneally injected (100 µg/kg/day) into HFD+C (0.01% CAP)-treated mice (*n* = 6/group) for 4 weeks (14). Mice were then subjected to GTT and analysis for markers of obesity, bacterial translocation, metabolic endotoxemia, and systemic CLGI.

### Statistical analyses.

Statistical analysis was performed using GraphPad Prism 6.01 (GraphPad Software, Inc., San Diego, CA) unless otherwise specified. Experiments with two groups were analyzed with Student’s *t* tests, and those with more than two groups were analyzed with one-way analysis of variance (ANOVA) with *post hoc* Bonferroni’s multiple comparison tests. Data were natural logarithm transformed to normalize their distributions if the values had a skewed distribution. Correlation-type principal-component analysis (PCA) (XLSTAT software) was applied to the correlation between groups and findings. Linear discriminant analysis effect size (LEfSe) uses a nonparametric Wilcoxon sum-rank test followed by LDA analysis to measure the effect size of each abundant taxon, and two filters (*P* < 0.05 and LDA score of >2) were applied to the present features. A *P* value of <0.05 was considered statistically significant.

Details of the rest of the materials and methods are provided in Text S1 in the supplemental material.

10.1128/mBio.00470-17.1 TEXT S1  Materials and methods. Download TEXT S1, DOCX file, 0.1 MB. Copyright © 2017 Kang et al. 2017 Kang et al. This content is distributed under the terms of the Creative Commons Attribution 4.0 International license.

### Accession number(s).

Sequencing data for the 16S rRNA sequences have been deposited in the SRA database under GenBank accession no. SRP099024.

10.1128/mBio.00470-17.9 TABLE S1  Primer sequences. Download TABLE S1, DOCX file, 0.1 MB. Copyright © 2017 Kang et al. 2017 Kang et al. This content is distributed under the terms of the Creative Commons Attribution 4.0 International license.

10.1128/mBio.00470-17.10 TABLE S2  Primers for amplifying target bacteria and butyryl-coenzyme A (CoA) transferase genes with qPCR. Download TABLE S2, DOCX file, 0.1 MB. Copyright © 2017 Kang et al. 2017 Kang et al. This content is distributed under the terms of the Creative Commons Attribution 4.0 International license.
