# Erratum for Kang et al., “Gut Microbiota Mediates the Protective Effects of Dietary Capsaicin against Chronic Low-Grade Inflammation and Associated Obesity Induced by High-Fat Diet”

**DOI:** 10.1128/mBio.00900-17

**Published:** 2017-07-05

**Authors:** Chao Kang, Bin Wang, Kanakaraju Kaliannan, Xiaolan Wang, Hedong Lang, Suocheng Hui, Li Huang, Yong Zhang, Ming Zhou, Mengting Chen, Mantian Mi

**Affiliations:** a Research Center for Nutrition and Food Safety, Institute of Military Preventive Medicine, Third Military Medical University, Chongqing Key Laboratory of Nutrition and Food Safety, Chongqing Medical Nutrition Research Center, Chongqing, People’s Republic of China; b Laboratory for Lipid Medicine and Technology, Department of Medicine, Massachusetts General Hospital and Harvard Medical School, Boston, Massachusetts, USA

## ERRATUM

Volume 8, no. 3, https://doi.org/10.1128/mBio.00470-17, 2017. The CB_1_ expression of the HFD and HFD+C groups were mistakenly switched in Fig. S7A in the supplemental material (our discussion of the results in the Results section and in the figure legend is accurate). We apologize for not detecting and correcting this error before publication. The file for Fig. S7 that contains the corrected labels has been replaced online.

