# Supplementary material for: Gut Microbiota Mediates the Protective Effects of Dietary Capsaicin against Chronic Low-Grade Inflammation and Associated Obesity Induced by High-Fat Diet
Source: mBio. 2017 May 23;8(3):e00470-17. doi: 10.1128/mBio.00470-17 (PMC5442453; doi:10.1128/mBio.00470-17)
Supplement: FIG S1 [file mbo003173307sf1.pdf]

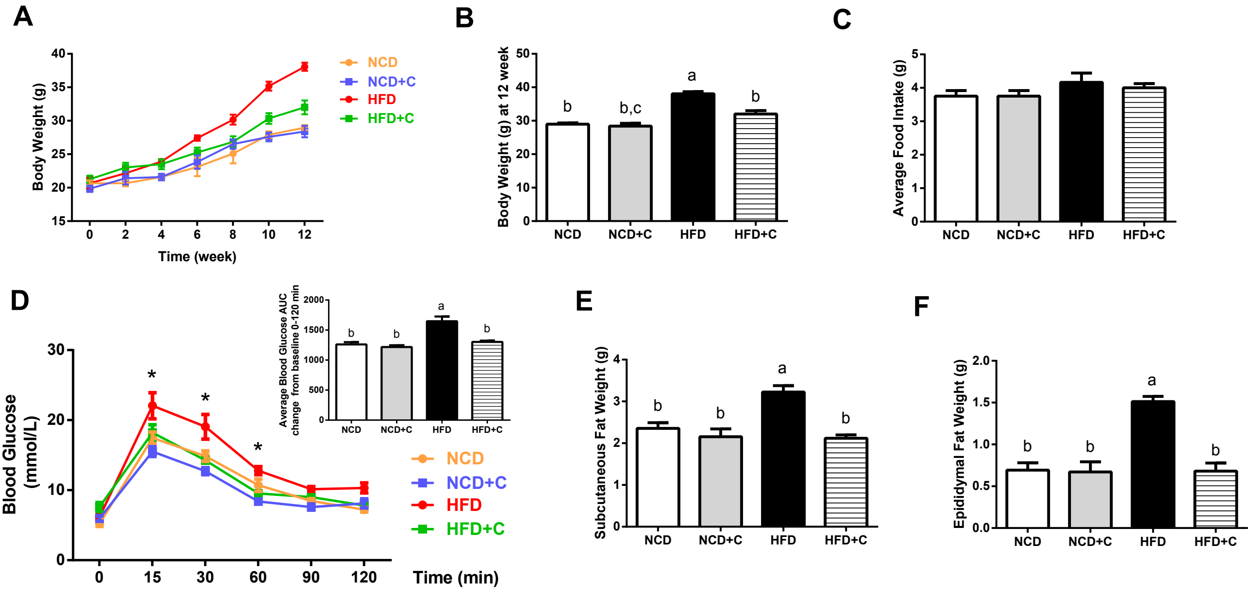

FIG S1. CAP reduced body weight, glucose intolerance and systemic low grade inflammation. Time course of body weight from mice a normal chow diet (NCD) and a high-fat diet (HFD) both with or without CAP supplementation over 12 weeks. (N = 6/group, A). The effects of dietary CAP on body weight (B), food intake(C), glucose tolerance (D), subcutaneous (E) and epididymal (F) adipose fat tissue weight are shown. Data are expressed as the mean± SEM. Data with different superscript letters are significantly different (P < 0.05) using ANOVA with a *post hoc* Bonferroni's multiple comparison test.
