# Supplementary material for: Gut Microbiota Mediates the Protective Effects of Dietary Capsaicin against Chronic Low-Grade Inflammation and Associated Obesity Induced by High-Fat Diet
Source: mBio. 2017 May 23;8(3):e00470-17. doi: 10.1128/mBio.00470-17 (PMC5442453; doi:10.1128/mBio.00470-17)
Supplement: FIG S2 [file mbo003173307sf2.pdf]

A

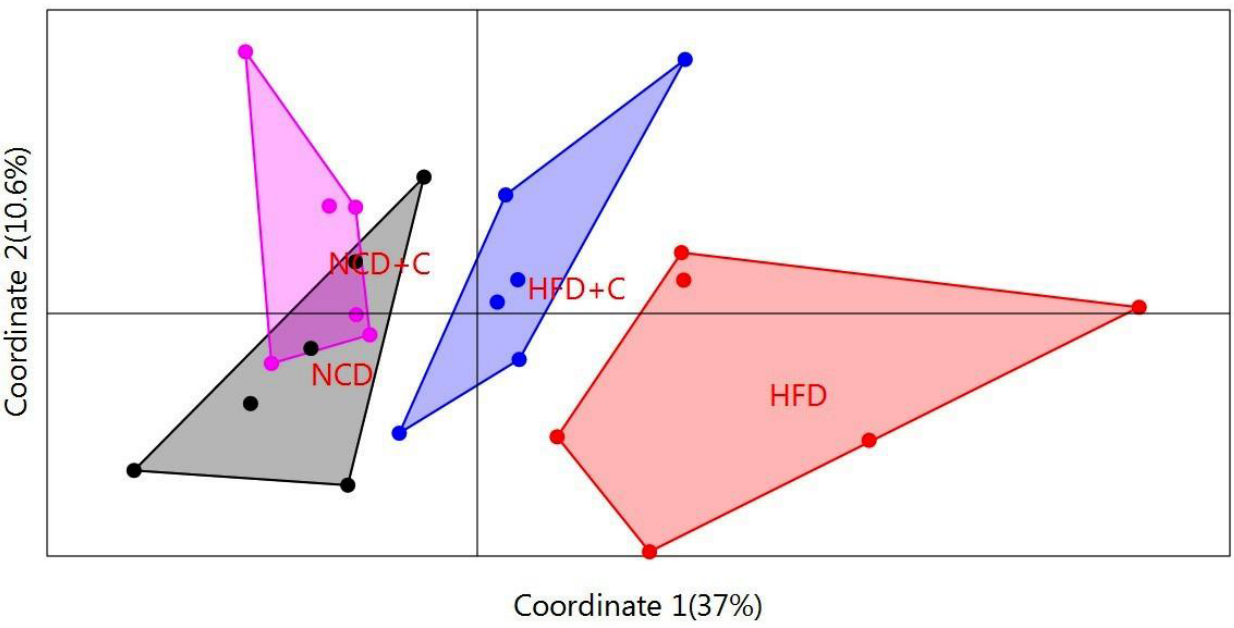

B

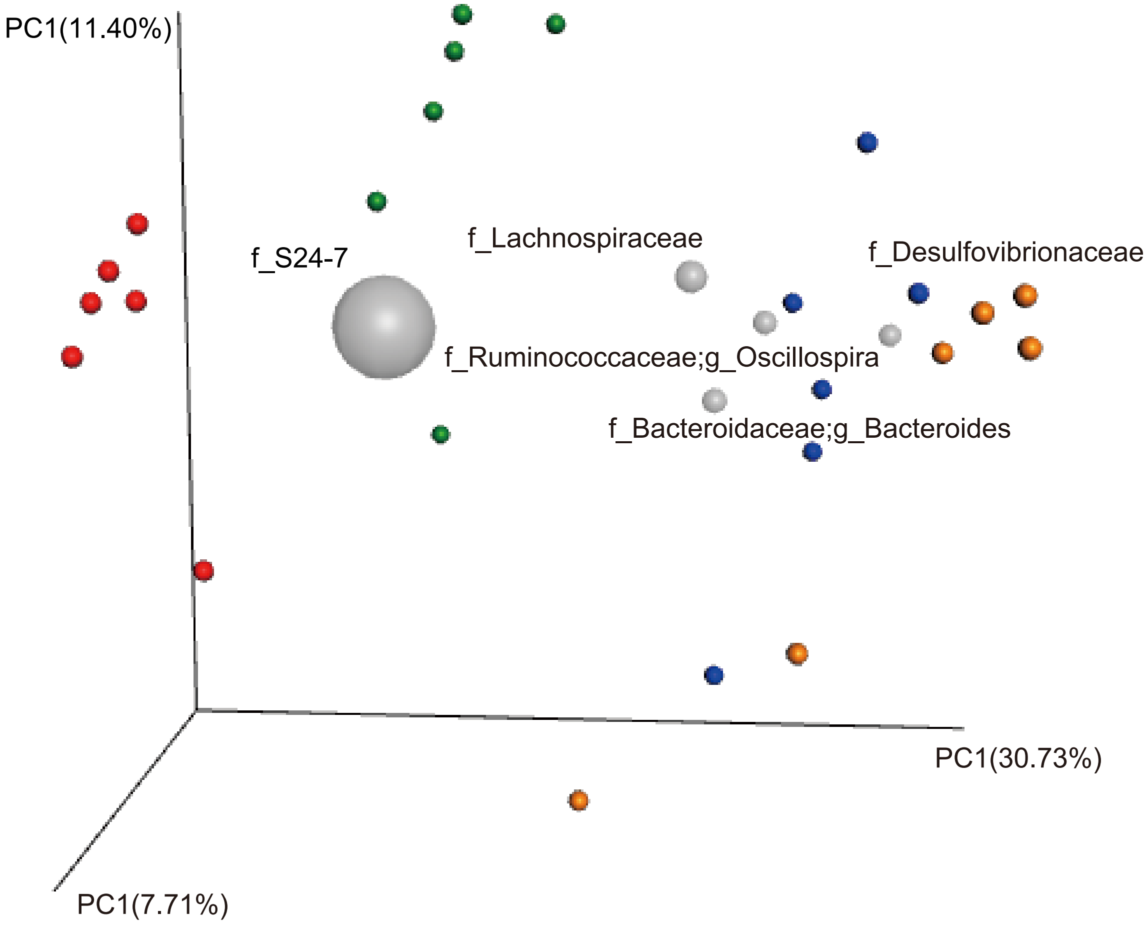

FIG S2. CAP beneficially alters gut microbiota. PCoA analysis using Jaccard index (A). PCoA analysis using Unweighted UniFrac distance (B). PC; principal coordinate.
