# Supplementary material for: Gut Microbiota Mediates the Protective Effects of Dietary Capsaicin against Chronic Low-Grade Inflammation and Associated Obesity Induced by High-Fat Diet
Source: mBio. 2017 May 23;8(3):e00470-17. doi: 10.1128/mBio.00470-17 (PMC5442453; doi:10.1128/mBio.00470-17)
Supplement: FIG S3 [file mbo003173307sf3.pdf]

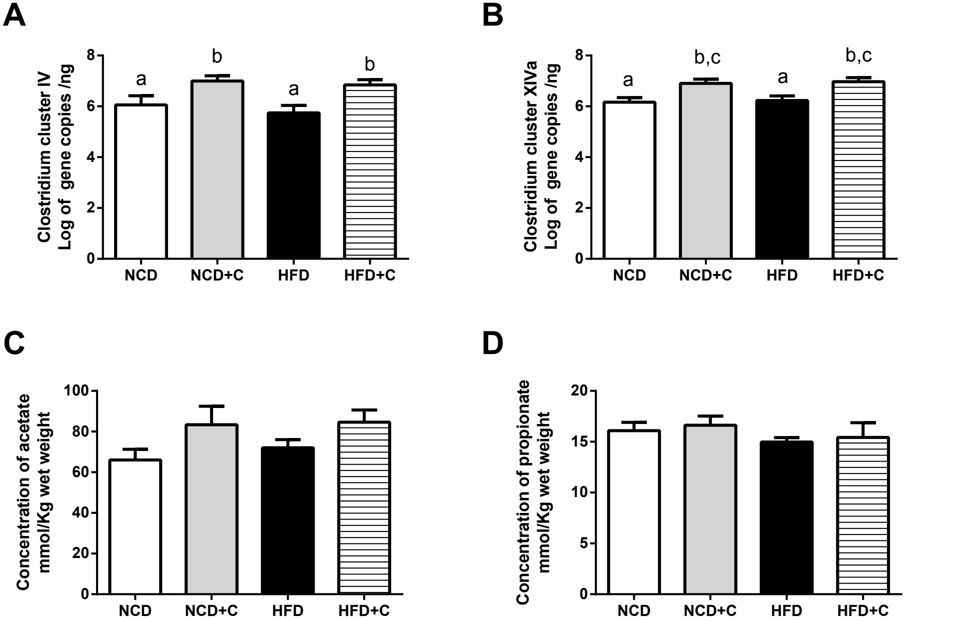

FIG S3. CAP alters the abundance of cecal butyrate-producing bacteria and fecal SCFA concentrations. qPCR analysis showing the abundance (gene copies) of *Clostridium cluster IV* (A) and *Clostridium cluster XIVa* (B). Fecal concentration of acetate (C) and propionate (D). Data are expressed as mean ± SEM. Data with different superscript letters are significantly different ($P < 0.05$) using one-way ANOVA with a *post hoc* Bonferroni's multiple comparison test.
