# Supplementary material for: Gut Microbiota Mediates the Protective Effects of Dietary Capsaicin against Chronic Low-Grade Inflammation and Associated Obesity Induced by High-Fat Diet
Source: mBio. 2017 May 23;8(3):e00470-17. doi: 10.1128/mBio.00470-17 (PMC5442453; doi:10.1128/mBio.00470-17)
Supplement: FIG S4 [file mbo003173307sf4.pdf]

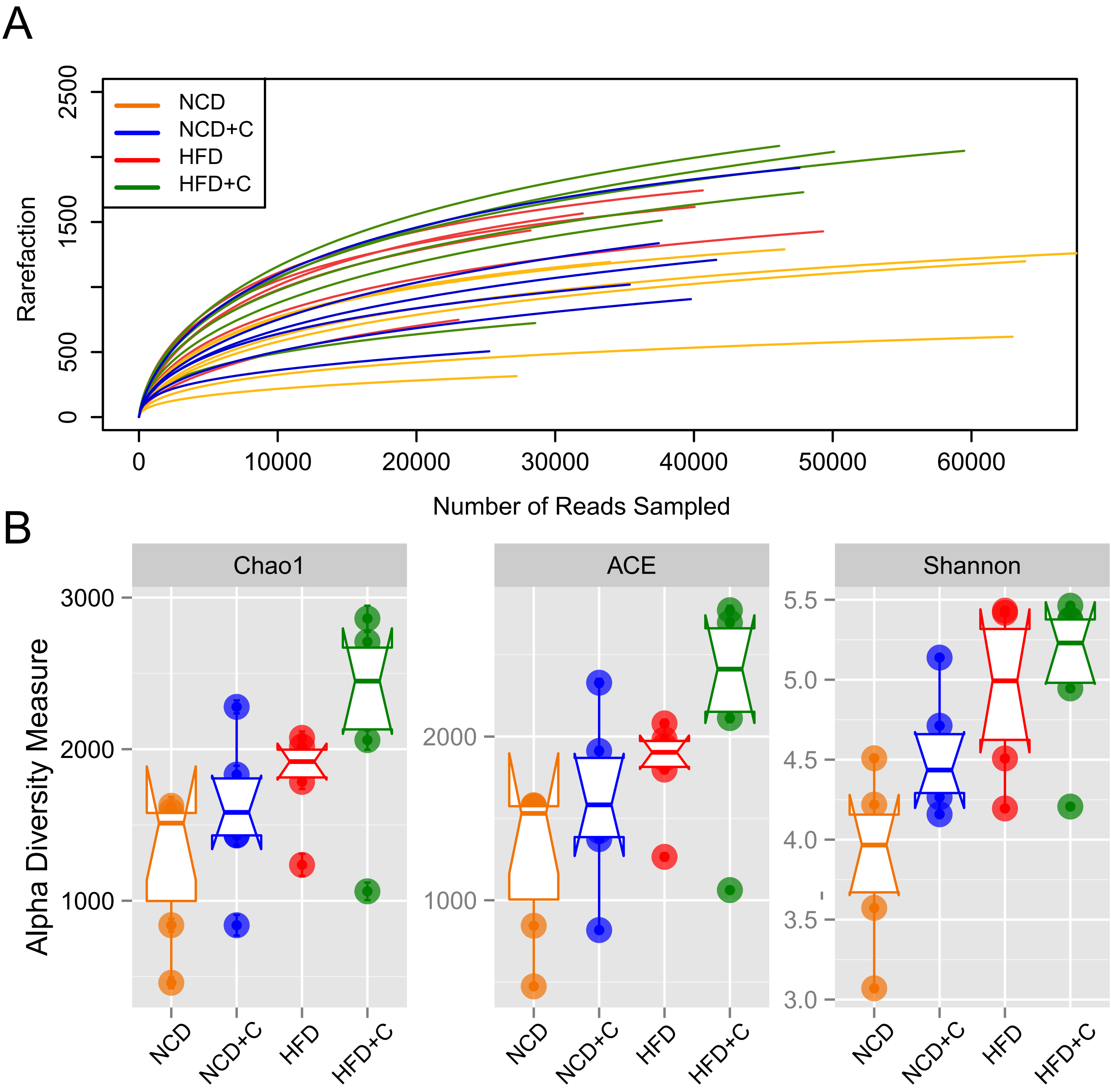

FIG S4. CAP beneficially alters gut microbiota. Rarefaction curves of sequencing samples (A) and α-diversity (B) of gut microbial communities assessed by Chao1, ACE and Shannon α-diversity index, respectively (data are presented as median with inter-quartile range).
