# Supplementary material for: Gut Microbiota Mediates the Protective Effects of Dietary Capsaicin against Chronic Low-Grade Inflammation and Associated Obesity Induced by High-Fat Diet
Source: mBio. 2017 May 23;8(3):e00470-17. doi: 10.1128/mBio.00470-17 (PMC5442453; doi:10.1128/mBio.00470-17)
Supplement: FIG S5 [file mbo003173307sf5.pdf]

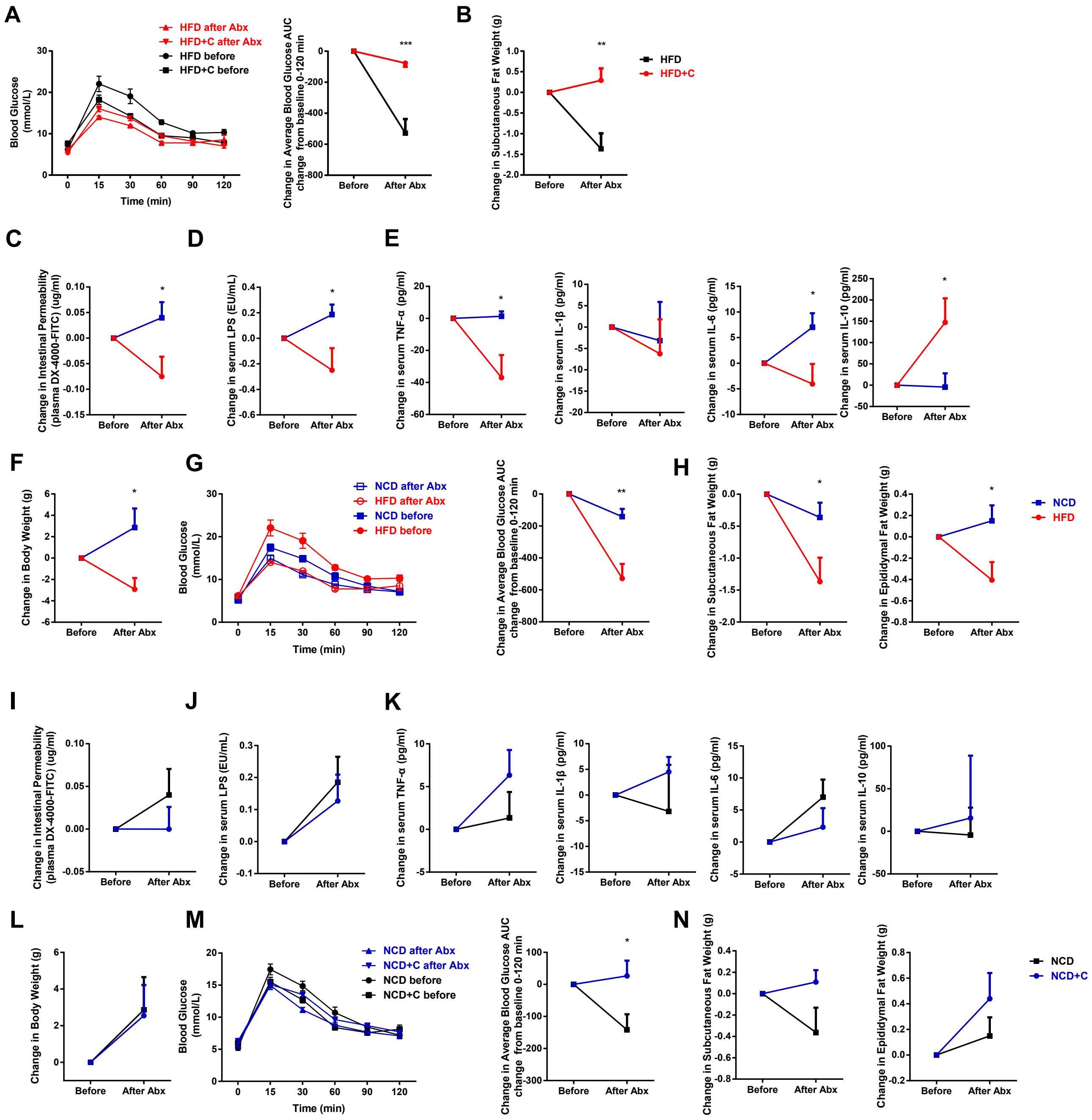

FIG S5. Gut microbiota mediates the beneficial effects of CAP on chronic inflammation and obesity. WT mice (n = 6/group) received HFD ± CAP and NCD ± CAP for 12 weeks and then treated with antibiotic (abx) cocktail for 6 weeks. Data were analyzed before and after Abx supplementation. 1) HFD vs. HFD+C; Markers of obesity and insulin resistance (A-B). 2) NCD vs. HFD; metabolic endotoxemia (ME) (C-D), chronic low grade inflammation (CLGI) (E) and obesity/insulin resistance (F-H). 3) NCD vs. NCD+C; ME (I-J), CLGI (K) and obesity/insulin resistance (L-N). Data are expressed as mean ± SE; Unpaired two-tailed student T-test. *$P < 0.05$; **$P < 0.01$;***$P < 0.001$.
