# Supplementary material for: Gut Microbiota Mediates the Protective Effects of Dietary Capsaicin against Chronic Low-Grade Inflammation and Associated Obesity Induced by High-Fat Diet
Source: mBio. 2017 May 23;8(3):e00470-17. doi: 10.1128/mBio.00470-17 (PMC5442453; doi:10.1128/mBio.00470-17)
Supplement: FIG S6 [file mbo003173307sf6.pdf]

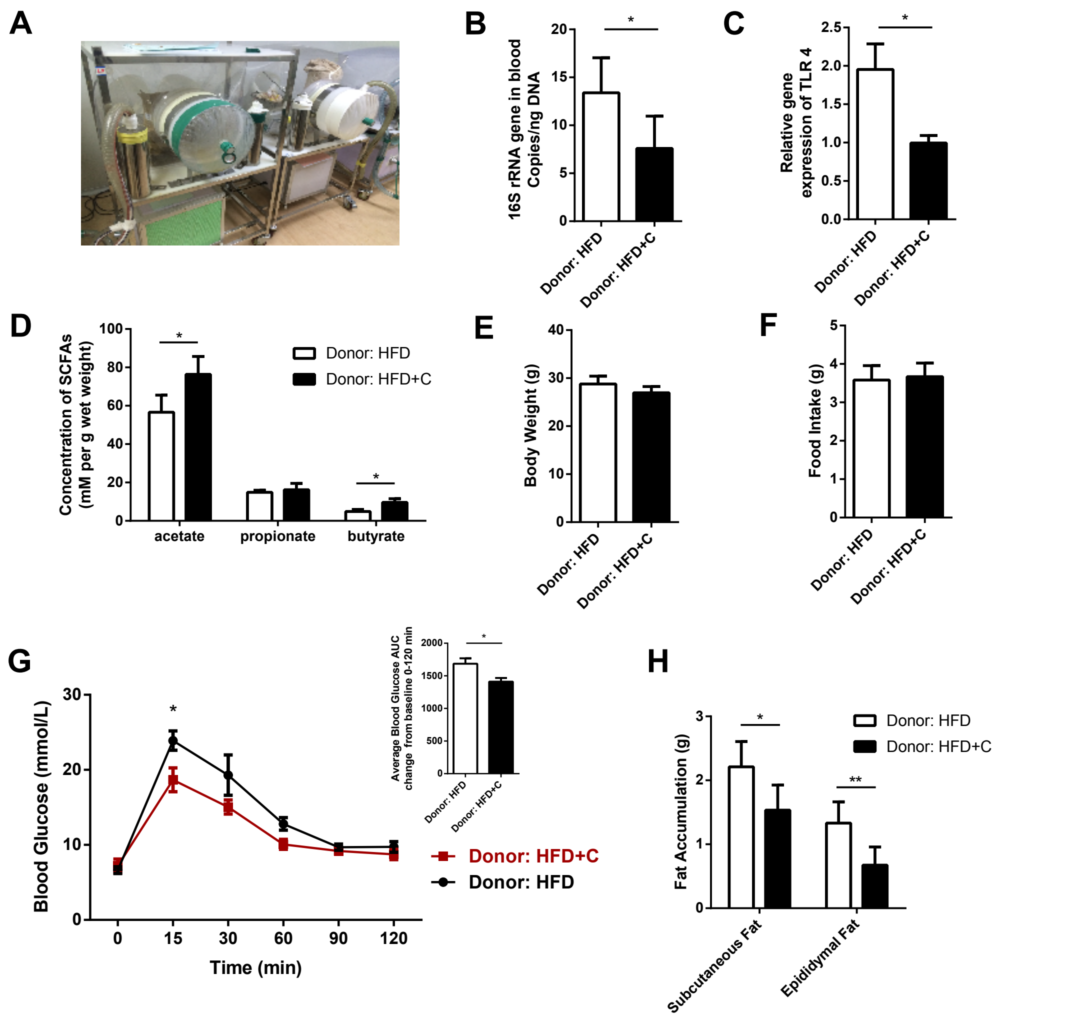

FIG S6. Anti obesity effects of CAP microbiota is transferable to germ-free mice. Gnotobiotic isolator (A). Bacterial DNA load in blood (B), adipose tissue TLR4 mRNA levels (C), fecal SCFA concentrations (D), Body weight (E), Food intake (F), glucose tolerance (G) and fat accumulation (H) of germ-free mice 14 days following transplant of microbiota from HFD-fed mice with or without CAP supplementation. Data are expressed as mean ± SEM. *P < 0.05 and **P < 0.01 using unpaired two-tailed Student's t-test.
