# Supplementary material for: Gut Microbiota Mediates the Protective Effects of Dietary Capsaicin against Chronic Low-Grade Inflammation and Associated Obesity Induced by High-Fat Diet
Source: mBio. 2017 May 23;8(3):e00470-17. doi: 10.1128/mBio.00470-17 (PMC5442453; doi:10.1128/mBio.00470-17)
Supplement: FIG S7 [file mbo003173307sf7.pdf]

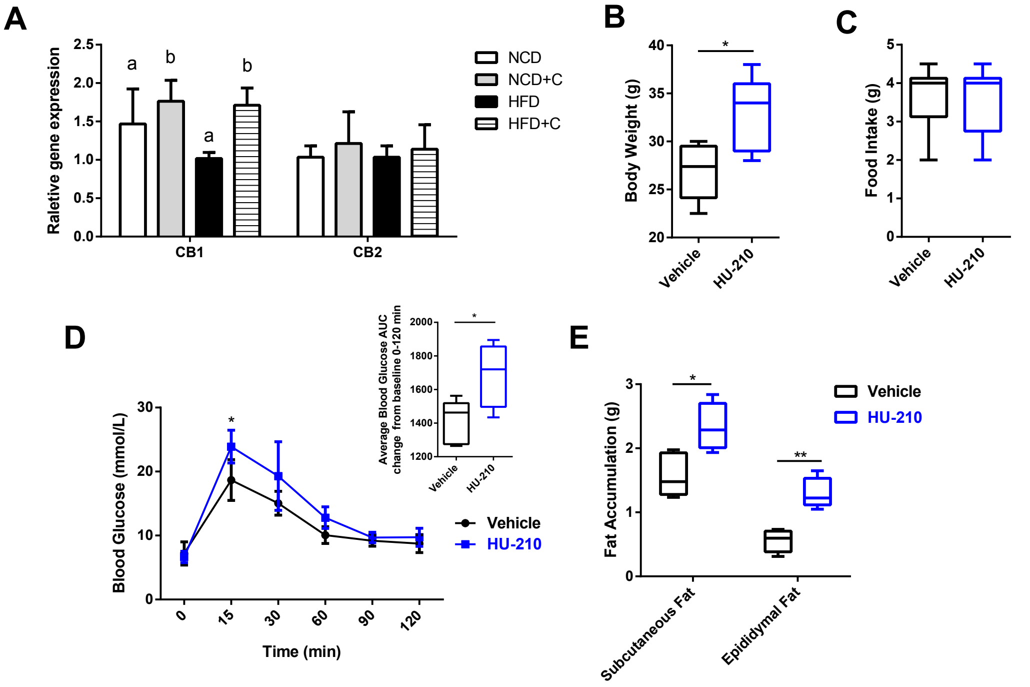

FIG S7. CAP down regulated CB1 receptor involves in the beneficial effects of CAP on obesity. mediated the benefits of CAP treatment. CB1 expression among mice fed with or without CAP treatment (A). A CB1-specific agonist (HU-210) was administered by orally gavaged to mice fed a HFD+CAP for 4 weeks. Activation of CB1 eliminated the beneficial effects of CAP treatment on body weight (B), food intake (C), OGTT (D) and fat accumulation (E). Data are expressed as the mean ± SEM. *$P < 0.05$ and **$P < 0.01$ using unpaired two-tailed Student's t-test. Data with different superscript letters are significantly different ($P < 0.05$) using ANOVA with a *post hoc* Bonferroni's multiple comparison test.
