# Supplementary material for: Gut Microbiota Mediates the Protective Effects of Dietary Capsaicin against Chronic Low-Grade Inflammation and Associated Obesity Induced by High-Fat Diet
Source: mBio. 2017 May 23;8(3):e00470-17. doi: 10.1128/mBio.00470-17 (PMC5442453; doi:10.1128/mBio.00470-17)
Supplement: TEXT S1 [file mbo003173307s1.docx]

**TextS1. Materials and Methods**

**Oral glucose tolerance test (OGTT)**

Mice were fasted overnight and glucose (2.0 g/kg body weight) was administered by oral gavage. Small blood samples (microliters) were drawn from the tip of the incised tail at 0, 15, 30, 60, 90 and 120 min to measure blood glucose levels with a glucose meter (Roche Diagnostics, Switzerland). Glucose tolerance was assessed by calculating the incremental area under the curve (AUC) of each GTT.

**Quantitative RT-PCR**

Total RNA was isolated from colon or epididymal white adipose tissues after processing with TRIzol reagent (Invitrogen Life Technologies, Grand Island, NY) following the manufacturer’s instructions. RNA concentrations and purity were estimated by determining the A260/A230 ratio with a Nanodrop 1000 spectrophotometer (Nanodrop Technologies, Wilmington, USA). Reverse transcription of mRNA was performed using the cDNA Synthesis Kit (Bio-Rad). PCR was carried out using SYBR Green with qTOWER 2.2 (Analytik Jena, Germany). Each sample was processed in triplicate and normalized to GAPDH or TBP by the 2^-ΔΔCT^ method, and the values were expressed relative to those of the control group. The primers sequences are shown in **Table S1.**

**Biochemical analyses and cytokine measurements**

Plasma LPS concentrations were determined using a ToxinSensor Chromogenic Limulus Amebocyte Lysate (LAL) Endotoxin Assay Kit (GenScript) as previous reported ([1](#_ENREF_1)). Briefly, Samples were diluted 1:20 and heated for 10 min at 70 °C. LAL reagents were added to plasma and incubated at 37 °C for 45 min, and the absorbance was read at 545 nm. A spiked control at 0.45 EU/ml was performed for each sample to check that no significant inhibition or activation occurred. ELISA kits were used to analyze the plasma levels of IL-1β, IL-6, IL-10 and TNF-a (R&D Systems, USA)

**Gut permeability assays**

Mice fasted for 4 hours were orally administered with 4000-Da FITC-dextran (46944; Sigma-Aldrich, 600 mg/kg body weight, 125 mg/mL). After 4 hours, the blood samples were collected in EDTA-Coated blood collection tubes and then plasma was separated by centrifuging at 6,000 rpm for 10 minutes at 4°C. Then, the plasma was diluted with same volume of PBS and analyzed for FITC-dextran by using a fluorospectrophotometer with an excitation wavelength of 485 nm and an emission wavelength of 535 nm as previously described ([2](#_ENREF_2)). Plasma FITC-dextran concentration was calculated from a standard curve generated by serial dilution of FITC-dextran in PBS (0–12.5 µg/mL).

**Bacteria fluorescence in situ hybridization (FISH)**

The FISH method was adapted from previous publication ([3](#_ENREF_3)). Briefly, tissue sections were deparaffinized with xylene and rehydrated through an ethanol gradient (95%, 10 min; 90%, 10 min) to water. The sections were then incubated with a universal bacterial probe directed against the 16S rRNA gene (EUB338: [Cy3]-GCTGCCTCCCGTAGGAGT-[AmC7~Q+Cy3es]) at 60 °C for 3 hours. The NON EUB 338 probe was applied as a negative control probe to recognize non-specific binding of the fluorochrome. The slides were washed and counter-stained by DAPI. The images were acquired using Zeiss LSM 700 confocal microscope with Zen 2012 software (blue edition).

**qPCR analysis of total and butyrate-producing bacteria**

Quantitative Real-Time PCR (qPCR) was used to quantify the total (16S rRNA copies) and butyrate-producing (Butyryl-Coenzyme A transferase genes, 16S rRNA copies of *Clostridium cluster XIVa* and *Clostridium cluster IV*) bacteria in the genomic DNA extracted from samples. Frozen cecal contents and whole blood samples were thawed at 4 °C. The total amount of genomic DNA was isolated from 200 mg of cecal contents and 50 µL of whole blood using QIAamp DNA Stool Mini Kit (QIAGEN, Hilden, Germany) and QIAamp Mini Kit respectively following the manufacturer’s instructions ([4](#_ENREF_4)). Standard curves for quantification consisted in ten-fold serial dilutions in the range of 10^8^ to 10^0^ copies of target 16S rRNA genes or CoA transferase genes from reference strains, amplified with primers listed in **Table S2**. qPCR was performed with a MyiQ single color Real-Time PCR detection system (Bio-Rad) with SYBR Real time PCR Kit (Takara, Japan). All measurements were performed in duplicates.

**Gut microbiome sequencing**

*1) 16S rDNA amplification and Illumina MiSeq sequencing.*  The samples were stored at -80 °C until genomic DNA (gDNA) extraction using the QIAamp DNA Stool Mini Kit (Qiagen, Hilden, Germany). The gDNA concentration and purity were estimated using a Nanodrop 1000 spectrophotometer. The bacterial 16S rRNA gene sequences of the fecal gDNA samples were PCR ampliﬁed using primers binding to the V3-V4 region, and the resulting amplicons were cleaned, quantified and sequenced on the Illumina MiSeq platform (Illumina, San Diego, CA, USA) with paired-end 300-nucleotide reads.

*2) Bioinformatics analyses.*  The raw data were then ﬁltered and demultiplexed using QIIME (v.1.8.0) as suggested ([5](#_ENREF_5)). With 97% identity, they were binned into operational taxonomic units (OTUs) and matched to entries in the SILVA 106 at an 80% confidence level. The reads that did not match a SILVA sequence with a 97% or greater sequence identity were discarded. The Chao1, Abundance-based Coverage Estimator (ACE) and Shannon α-diversity indexes were calculated and rarefaction curve analysis was performed by using Mothur (v.1.30.1) as suggested ([6](#_ENREF_6)). Ordinations are the dimensional-reduction techniques which are commonly used to visualize complex relationships between communities. Dimensional reduction of the Bray-Curtis distance between microbiome samples using Principal Coordinate Analysis (PCoA) ordination method (XLSTAT) was done and the significant differences among groups were tested with Permutational Analysis of Variance (PERMANOVA), a multivariate non parametric one way ANOVA, which utilizes the sample-to-sample distance matrix directly. Top 7 taxa which are primarily responsible for an observed difference between groups were identified by SIMPER (Similarity Percentage) method and their contribution to groups (between and within groups) were analyzed using Principal Component variance-covariance type ordination (XLSTAT software) method. Differential expression of taxon were identified (non parametric ANOVA with Benjamini-Hochberg false discovery rate correction; *P* <0.05) and those with *P* <0.05 (41 taxa’s) were grouped and their relative abundance were shown by heat map with hierarchal clustering (HCN) analysis ([7](#_ENREF_7)). Microbiota based biomarker discoveries were done with the Linear Discriminant Analysis Effect Size (LEfSe) and the LDA scores derived from LEfSe analysis ([8](#_ENREF_8)) were used to show the relationship between taxon using a cladogram (circular hierarchical tree) of significantly increased or decreased bacterial taxa in the gut microbiome of four groups (FDR <0.05). Levels of the cladogram represent, from the inner to outer rings, phylum, class, order, family, and genus. Color codes indicate the groups, and letters indicate the taxa that contribute to the uniqueness of the corresponding groups at an LDA of >2.0.

*3) Putative metagenome identification.* Microbial functions were predicted using 16S ribosomal RNA sequencing and phylogenetic reconstruction of unobserved states (PICRUSt) software (version 1.0.0) as suggested ([9](#_ENREF_9)). The predicted genes and functions were aligned to KEGG database (version 66.1, May 1, 2013). STAMP (version 2.0.9) was utilized to determine significant putative KEGG orthologs and pathway analyses with two-side Welch’s t-test including two filters (*P* value < 0.05 and effect size > 0.2) applied to present features ([10](#_ENREF_10)).

**SCFA measurement**

Fatty acid analysis was conducted using an Agilent 6890N GC system (Agilent Technologies, PA, USA) and performed as previous described ([11](#_ENREF_11)). Briefly, fecal pellets from each mouse were weighted and homogenized in 1 mL deionized water for 3min. The pH value of the suspension was adjusted to 2–3 and suspension was subsequently transferred into a polypropylene tube and centrifuged for 20 min at 3,000g, yielding a clear supernatant. 2-ethylbutyric acid (TEBA) was used as the internal standard and added into the supernatant at a final concentration of 1mM.

Reference

1. Kaliannan K, Wang B, Li XY, Kim KJ, Kang JX. 2015. A host-microbiome interaction mediates the opposing effects of omega-6 and omega-3 fatty acids on metabolic endotoxemia. Sci Rep 5:11276.

2. Cani PD, Bibiloni R, Knauf C, Waget A, Neyrinck AM, Delzenne NM, Burcelin R. 2008. Changes in gut microbiota control metabolic endotoxemia-induced inflammation in high-fat diet-induced obesity and diabetes in mice. Diabetes 57:1470-81.

3. Vaishnava S, Yamamoto M, Severson KM, Ruhn KA, Yu X, Koren O, Ley R, Wakeland EK, Hooper LV. 2011. The antibacterial lectin RegIIIγ promotes the spatial segregation of microbiota and host in the intestine. Science 334:255-258.

4. Caesar R, Tremaroli V, Kovatcheva-Datchary P, Cani PD, Backhed F. 2015. Crosstalk between Gut Microbiota and Dietary Lipids Aggravates WAT Inflammation through TLR Signaling. Cell Metabolism 22:658-668.

5. Caporaso JG, Kuczynski J, Stombaugh J, Bittinger K, Bushman FD, Costello EK, Fierer N, Pena AG, Goodrich JK, Gordon JI, Huttley GA, Kelley ST, Knights D, Koenig JE, Ley RE, Lozupone CA, McDonald D, Muegge BD, Pirrung M, Reeder J, Sevinsky JR, Turnbaugh PJ, Walters WA, Widmann J, Yatsunenko T, Zaneveld J, Knight R. 2010. QIIME allows analysis of high-throughput community sequencing data. Nat Methods 7:335-6.

6. Schloss PD, Westcott SL, Ryabin T, Hall JR, Hartmann M, Hollister EB, Lesniewski RA, Oakley BB, Parks DH, Robinson CJ, Sahl JW, Stres B, Thallinger GG, Van Horn DJ, Weber CF. 2009. Introducing mothur: open-source, platform-independent, community-supported software for describing and comparing microbial communities. Appl Environ Microbiol 75:7537-41.

7. Chevalier C, Stojanović O, Colin DJ, Suarez-Zamorano N, Tarallo V, Veyrat-Durebex C, Rigo D, Fabbiano S, Stevanović A, Hagemann S. 2015. Gut Microbiota Orchestrates Energy Homeostasis during Cold. Cell 163:1360-1374.

8. Segata N, Izard J, Waldron L, Gevers D, Miropolsky L, Garrett WS, Huttenhower C. 2011. Metagenomic biomarker discovery and explanation. Genome Biology 12:1-18.

9. Langille MGI, Zaneveld J, Caporaso JG, McDonald D, Knights D, Reyes JA, Clemente JC, Burkepile DE, Vega Thurber RL, Knight R, Beiko RG, Huttenhower C. 2013. Predictive functional profiling of microbial communities using 16S rRNA marker gene sequences. Nat Biotech 31:814-821.

10. Parks DH, Beiko RG. 2010. Identifying biologically relevant differences between metagenomic communities. Bioinformatics 26:715-721.

11. Zhao G, Nyman M, Jonsson JA. 2006. Rapid determination of short-chain fatty acids in colonic contents and faeces of humans and rats by acidified water-extraction and direct-injection gas chromatography. Biomed Chromatogr 20:674-82.
