# Supplementary material for: Gut Microbiota Mediates the Protective Effects of Dietary Capsaicin against Chronic Low-Grade Inflammation and Associated Obesity Induced by High-Fat Diet
Source: mBio. 2017 May 23;8(3):e00470-17. doi: 10.1128/mBio.00470-17 (PMC5442453; doi:10.1128/mBio.00470-17)
Supplement: TABLE S1 [file mbo003173307st1.docx]

Table S1: Primers sequences

| **Name** | **Forward Sequence** | **Reverse Sequence** |
| --- | --- | --- |
| **ZO-1** | ACCCGAAACTGATGCTGTGGATAG | AAATGGCCGGGCAGAACTTGTGTA |
| **Occludin** | ATGTCCGGCCGATGCTCTC | TTTGGCTGCTCTTGGGTCTGTAT |
| **TLR 4** | CGCTTTCACCTCTGCCTTCACTACAG | ACACTACCACAATAACCTTCCGGCTC |
| **CB _1_** | CTGATGTTCTGGATCGGAGTC | TCTGAGGTGTGAATGATGATGC |
| **CB _2_** | TGACAAATGACACCCAGTCTTCT | ACTGCTCAGGATCATGTACTCCTT |
| **GAPDH** | GCATCCACTGGTGCTGCC | TCATCATACTTGGCAGGTTTC |
| **TBP** | ACCCTTCACCAATGACTCCTATG | TGACTGCAGCAAATCGCTTGG |
