# Supplementary material for: Gut Microbiota Mediates the Protective Effects of Dietary Capsaicin against Chronic Low-Grade Inflammation and Associated Obesity Induced by High-Fat Diet
Source: mBio. 2017 May 23;8(3):e00470-17. doi: 10.1128/mBio.00470-17 (PMC5442453; doi:10.1128/mBio.00470-17)
Supplement: TABLE S2 [file mbo003173307st2.docx]

Table S2: Primers for amplifying target bacteria and butyryl-coenzyme A (CoA) transferase genes with qPCR.

| **Target bacterial group** | **Primers** | **Primer Sequences** | | **References** |  |
| --- | --- | --- | --- | --- | --- |
| **Total bacteria** | Uni-1 (331F) | 5’-TCCTACGGGAGGCAGCAGT-3’ | ([1](#_ENREF_1)) | | |
|  | Uni-2 (797R) | 5’-GGACTACCAGGGTATCTAATCCTGTT-3’ |  |  |  |
| **Butyrate producing bacteria** | BCoATscrF | 5’-GCIGAICATTTCACITGGAAYWSITGGCAYATG-3’ | ([2](#_ENREF_2)) | | |
|  | BCoATscrR | 5’-CCTGCCTTTGCAATRTCIACRAANGC-3’ |  |  |  |
| **Clostridium cluster XIVa** | 195-F | 5’-GCAGTGGGGAATATTGCA-3’ | ([3](#_ENREF_3)) | | |
|  | Ccocc-R | 5’-CTTTGAGTTTCATTCTTGCGAA-3’ |  |  |  |
| **Clostridium cluster IV** | sg-Clept-F | 5’-GCACAAGCAGTGGAGT-3’ | ([3](#_ENREF_3)) | | |
|  | sg-Clept-R3 | 5’-CTTCCTCCGTTTTGTCAA-3’ |  |  |  |

**References**

1. Nadkarni MA, Martin FE, Jacques NA, Hunter N. 2002. Determination of bacterial load by real-time PCR using a broad-range (universal) probe and primers set. Microbiology 148:257-266.

2. Louis P, Flint HJ. 2007. Development of a semiquantitative degenerate real-time pcr-based assay for estimation of numbers of butyryl-coenzyme A (CoA) CoA transferase genes in complex bacterial samples. Appl Environ Microbiol 73:2009-12.

3. Hippe B, Zwielehner J, Liszt K, Lassl C, Unger F, Haslberger AG. 2011. Quantification of butyryl CoA:acetate CoA-transferase genes reveals different butyrate production capacity in individuals according to diet and age. FEMS Microbiol Lett 316:130-5.
